# The Press Bazaar in Aid of the London Hospital

**Published:** 1898-07-02

**Authors:** 


### THE PRESS BAZAAR IN AID OF THE LONDON HOSPITAL.

However much editors may differ in the opinions on politics they offer to their readers, the Press Bazaar has shown us that they are capable of joining forces, and, still further, that when combined they are irresistible. Indeed, were they to carry the public with them in politics to the same extent they do in charity, they would only have to sink their differences to render the Upper and Lower Houses cumbersome superfluities.

The Press Bazaar has, indeed, been a glorious success. From some time before the opening hour the Strand was hopelessly blocked, but the crush there was nothing to the block at the entrances, and still less to the crowd of well-dressed humanity inside. Indeed, so closely packed were people in the rooms of the Hotel Cecil that it is difficult to understand how they succeeded in reaching the stalls and making their purchases. Yet so many purchases did they make that in some cases stall-keepers were reduced to the satisfactory state of having to wonder how they could manage to replenish their stalls for the second day, and this by tea-time on the opening day! What the profits of the bazaar have been will probably not be announced until these lines are in print, but there is no doubt they are enormous, and will do much to ease the straitened finances of the London Hospital; and, in the meantime, we can warmly congratulate all who gave their assistance to the bazaar, either by purchasing, by giving, or by serving, on the satisfactory manner in which a most deserving charity will benefit by their generosity and exertions.

The bazaar was opened by the Princess of Wales, accompanied by the Duchess of York, at half-past two on Tuesday, and from that hour business was fast and furious. The Princess, with that practical interest in all good works which has endeared her to us all, set the example by making purchases from many of the stalls, an example which was most loyally followed.

So great was the crowd that it is difficult to give a general description of the appearance of the bazaar. The stalls in the main hall were all formed into tents, with draperies of yellow and white muslin, through which the electric light showed off the wares to the best advantage. So numerous were the stalls that it is impossible with the space at our disposal to give a detailed description of all, and we must confine ourselves to the few that the crowd enabled us to more closely inspect.

The *Artist* stall, superintended by Miss Trevor-Battye, occupied the space at the entrance to the refreshment room. It was most gracefully arranged with lace, pottery, and embroidery, while the surrounding walls were hung with many valuable water colours, which, we trust, found the purchasers their undoubted merit deserved.

The *Financial News*, undertaken by Mrs. H. H. Marks, showed jewellery, diamonds, specimens of gold ore, &c. The jewellery was not only good, but was most moderately priced, and the sale must have been most satisfactory.

The *Lady*, the *Queen*, and the *Gentlewoman* showed chiffons, fichus, and French hats; of the latter it is difficult for anyone but an expert to speak, but they were undoubtedly visions of beauty, and more than silent testimony was borne to their merits by a large and admiring crowd of ladies.

The *Morning Post* occupied the space at the foot of the stairs with a flower stall, superintended by Lady Tweedmouth, and it is difficult to imagine a more beautiful sight than the stall presented, festooned with light traceries of smilax and pink carnations, forming a bower for the lovely blossoms displayed.

The *Naval and Military Magazine* showed service knick-knacks and Wellington relics, presided over by Mrs. Arthur àBeckett. Small pieces of the *Victory*, tied up in red, white, and blue ribbon, met with a ready sale; while the Wellington relics were interesting. The stall-holders at this stall were appropriately dressed in naval and military costumes.

The *Jewish Chronicle*, superintended by Mrs. Nathaniel Cohen, had a most attractive stall of miscellaneous articles, and it is very satisfactory to know that numerous purchasers showed their appreciation of the pretty articles offered for sale.

The "Cartoons and Caricatures" of the *Westminster Gazette*, sold by Mrs. Spender, achieved a huge success, as, indeed, their excellence deserved, the political d'oyleys forming a specially attractive bait. It is more than satisfactory to be able to chronicle this success, for the interest taken in the bazaar by the *Westminster* and the labours of Mrs. Spender went very far to make the bazaar what it was.

The Hospital stall, superintended by Lady Burdett and Mrs. Woolrych Perowne, ably assisted by Lady Agnes Anderson, Lady Muriel Watkins, Lady Granville Gordon, the Hon. Emily Kinnaird, Lady Thomas, Mrs. Carr-Gomm, Mrs. Whipple, Miss Armyn Gordon, Miss Kennaway, Miss Burdett, the Misses Hare, Miss Pritchard, Miss Probyn, Miss B. Willoughby, and others, achieved a success of which we are both proud and gratified. As our readers are aware, the stall was devoted to fruit; and, indeed, from the generous way in which our appeals for presents of fruit were answered, we have good reason to know the fact was widely known. To thank all our kind friends here would be impossible. Our acknowledgments will be found elsewhere. The lavish way in which H.R.H. the Prince of Wales, Lord Rothschild, Mr. Leopold de Rothschild, Mrs. Watney, and Mr. Pierrepont Morgan especially responded to our requests enabled us to present such a show of fruit as, we think, has seldom been seen before. Peaches, nectarines, grapes, strawberries, cherries, pineapples—every fruit, English and foreign, in season or out of season, and all in the most perfect condition —were piled in artistic profusion on our stall. Baskets of every size and shape, baskets of peaches, baskets of grapes — baskets of various fruits, heaped in artistic masses — poured in on us from all quarters, and we are happy to say also met with a ready sale. Beautiful as the masses of fruit were in themselves, the tasteful arrangement of the stall by the willing workers who laboured in the morning made the *tout ensemble* still more beautiful. To all our helpers we can only offer our grateful thanks. We feel sure that the knowledge of the success they enabled us to attain is more to them than anything we can say. We must not, however, forget to mention our *pièce de resistance*—our Sandringham ship—crowded with fruit from stem to stern, from keel to top-gallants. Her harbour dues (the ridiculous sum of five guineas) were soon paid, and she hoisted the blue Peter very early the first day; thanks, however, to the kindly purchaser, she was left to adorn our stalls till late in the evening.

The hospitals gave us a ready means of disposing of such fruit as was left unsold each evening. A brisk trade was done in strawberries and cream. The latter was a present from Messrs. Dahl, of their pure double cream, which was whipped and served in tempting bowls. In spite of the generosity of the present the supply fell far short of the demand, owing to the popularity of the delicious cream. The splendid palms and artistic floral decoration was generously undertaken by Messrs. Piper, the well-known florists in Bishop's Road, Paddington. Mr. Piper spared no pains to assist in rendering The Hospital stall attractive. Some of our kindest friends were Mr. Nathanel Cohen, of the *Jewish Chronicle*, and the Hon. Sydney Holland. Mr. Cohen brought numerous friends to make purchases, and cheered and encouraged the weary stallholders by his kindly presence and help. Indeed, The Hospital can never forget the number of kind and generous friends who rallied round it.

Probably the most attractive feature of the bazaar was the room in which the "Press Bazaar News" was written, composed, edited, printed, and sold. It is described as the "dearest" little paper in the world, but with all due respect to the galaxy of talent by which it was brought out, we must cavil at the term "dearest." To our mind "dear" means that a price greater than the value of the article sold is charged; but when such a unique memento is offered for the price of a shilling the term is absurd, except in the affectionate sense. At any rate, we hope those who bought the paper will keep their copies for, say, ten years, then sell them and send what they fetch to the London Hospital. We shall be much surprised if they are not worth then half a guinea apiece.

Inside the enclosure leading editors composed, corrected, set the type, and in their shirt sleeves worked the printing machine. Outside the people stood waiting for the next edition to appear. On the wall hung trophies brought by war correspondents from all parts of the world, chief among them a standard with an Arabic inscription upside down—doubtless as a signal of distress because the printers could not turn out the paper rapidly enough to supply the clamouring public.

In the Victoria Hall, all the leading actors and actresses gave performances twice a day; in secluded spots the phonograph, the gramophone, and the electrophone gave entertainments as long as the bazaar was open.

For the inquisitive, Mesdames Albu and Cassandra and Signor Rupert told the past, the present, and the future. Indeed, the latter told us so much about ourselves that we came away with the idea that Signor Rupert had been acquainted with us since childhood, and a thorough belief in his prophecies for the future.

